# Two case reports of eosinophilic fasciitis with onset after immune checkpoint inhibitor cessation

**DOI:** 10.3389/fonc.2026.1613243

**Published:** 2026-03-04

**Authors:** Christopher F. Theriau, Nancy Maltez, Niloufar Hosseini, Stephanie Petkiewicz, Xinni Song, Michael Ong

**Affiliations:** 1 Division of Medical Oncology, Department of Medicine, The Ottawa Hospital Cancer Centre, Ottawa, ON, Canada; 2 Division of Rheumatology, Department of Medicine, The Ottawa Hospital, Ottawa, ON, Canada; 3 Division of Diagnostic and Molecular Pathology, Department of Laboratory Medicine, The Ottawa Hospital, Ottawa, ON, Canada

**Keywords:** case report, checkpoint inhibitors, eosinophilic fasciitis, melanoma, urothelial carcinoma

## Abstract

Immune checkpoint inhibitors (ICIs) are known to cause a wide spectrum of immune-related adverse events (irAEs). Among these, eosinophilic fasciitis (EF) is a rare, fibrosing disorder causing inflammatory infiltration of subcutaneous fat and fascia. It is characterized clinically by edema and subsequent induration and tightening of the skin and subcutaneous tissues. Several case reports have documented EF secondary to ICIs in patients on active treatment. Herein, we present two cases of delayed EF following treatment cessation with avelumab for metastatic urothelial carcinoma (Case 1) and following adjuvant nivolumab completion for stage IIIC melanoma (Case 2). Both patients had typical exam findings including erythema/edema of the extremities and trunk and diffuse thickening of subcutaneous fat and fascia, leading to severe respiratory restriction in Case 1. Both patients were diagnosed with EF by full-thickness skin biopsy showing sclerosis and lymphocytic infiltration of the subcutaneous fat and/or fascia. Only one of the two patients presented with definite eosinophilia. Both cases were treated with glucocorticoids and had early recurrence of symptoms post steroid taper, necessitating subsequent protracted steroid and steroid sparing agent use. Overall, we demonstrate the importance of considering delayed irAEs, specifically autoimmune fibrotic skin diseases even when ICI therapy has been discontinued. We underscore the need for collaboration between oncology and rheumatology as the scope of ICI treatments for cancer continues to expand.

## 1 Introduction

Immune checkpoint inhibitors (ICIs) are monoclonal antibodies used to treat many solid tumors. In targeting immune regulatory pathways, ICIs can inadvertently trigger a spectrum of immune-related adverse events (irAEs). Rheumatological irAEs are reported in 5%–22% of patients, and common arthralgias and arthritis are generally manageable (1). However, rare rheumatologic irAEs can pose significant diagnostic and therapeutic challenges. One rare entity is eosinophilic fasciitis (EF), an autoimmune fibrotic scleroderma mimic. EF is characterized by erythema and edema of the extremities and trunk, followed by diffuse collagenous thickening of the subcutaneous fat and fascia (2, 3). Physical exam findings of EF include peau d’orange skin typically sparing the hands and feet with a “woody induration” and a “groove sign”, while laboratory tests may reveal hypergammaglobulinemia and peripheral eosinophilia (2, 4). There are no widely accepted criteria for diagnosis, but proposed classification criteria include the Pinal-Fernandez and Jinnin criteria (4, 5). Management generally involves holding the inciting ICI and utilizing immunosuppressive agents such as corticosteroids, immunosuppressives, biologics, or a combination (6).

Several case reports have documented the association of ICI-induced EF with the use of pembrolizumab, nivolumab, atezolizumab, and avelumab weeks to several months after initiation of therapy (7, 8). In all these previous reports, patients were actively undergoing treatment when they developed EF. Herein, we present two cases of delayed EF after discontinuation of ICI therapy: one case at 1 month following cessation of avelumab for treatment of metastatic bladder cancer and the other case at 9 months after ceasing nivolumab for treatment of locally advanced melanoma.

## 2 Case presentation

### 2.1 Case 1

A previously healthy 81-year-old man with a past medical history of hypertension and benign prostatic hyperplasia on tadalafil, tamsulosin, and telmisartan-hydrochlorothiazide presented with intermittent gross hematuria with cystoscopy showing a large bladder mass. Transurethral resection of bladder tumor (March 2021) confirmed a high-grade muscle-invasive urothelial carcinoma. Evaluation by computed tomography (CT), magnetic resonance imaging (MRI), and bone scans demonstrated 4th/5th rib metastases without other distant metastases. He received cisplatin 67.5 mg/m^2^ (reduced for elderly status) plus gemcitabine 1,000 mg/m^2^ intravenously (IV) on day 1 and gemcitabine 1,000 mg/m^2^ IV on day 8 every 3 weeks for four cycles (May–July 2021). Repeat imaging after treatment showed stable rib metastases. He declined primary/oligometastatic radiotherapy in favor of ongoing systemic therapy and was switched to maintenance avelumab (10 mg/kg) IV every 2 weeks. Treatment was well tolerated without toxicity for 63 cycles (September 2021–April 2024) with clinical and radiographic stability.

**Figure 1:**
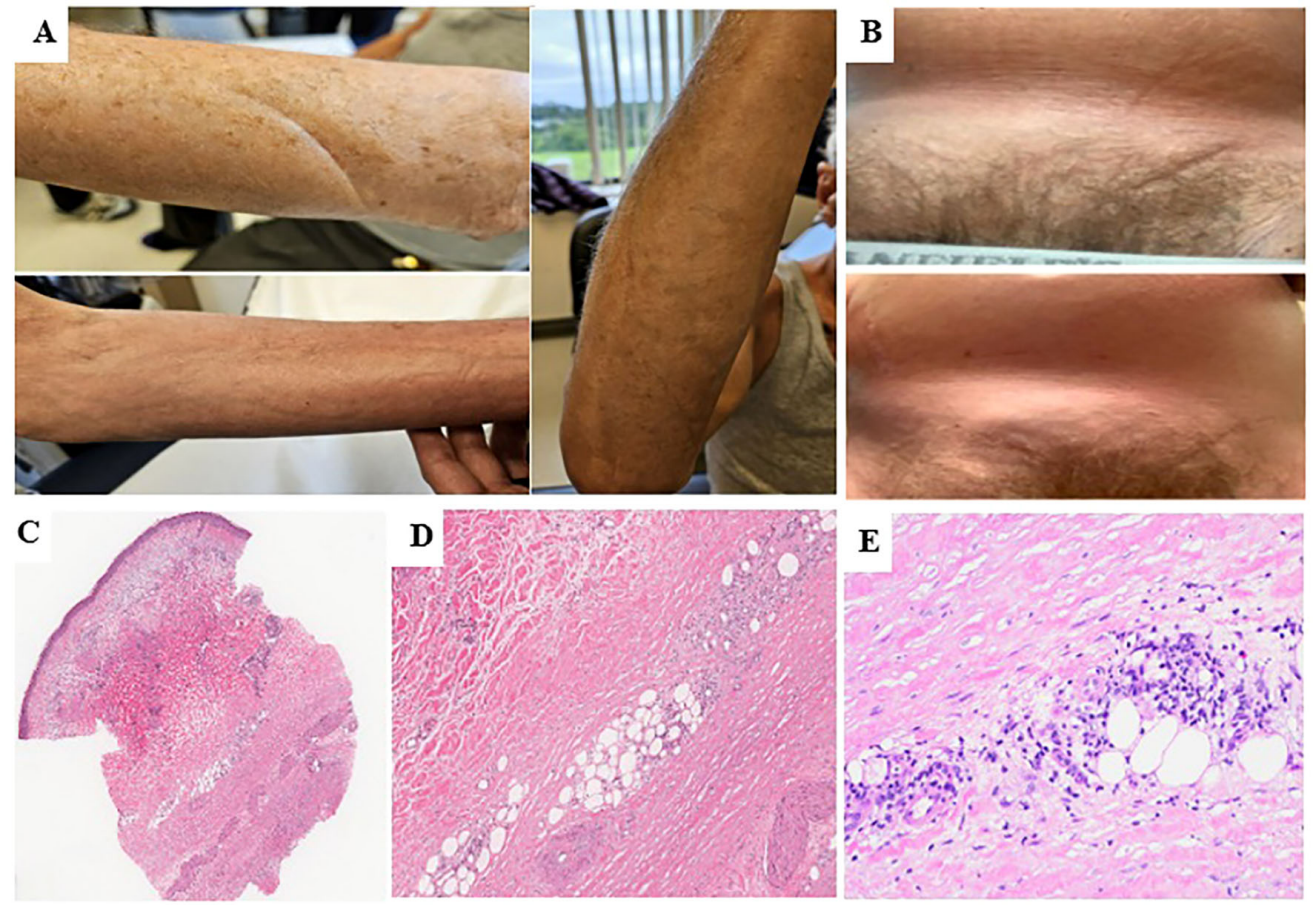
Physical exam findings and H&E staining for case 1- **(A)** Physical exam findings showing groove sign on BL forearms on initial assessment at presentation. **(B)** Late physical exam findings of abdominal wall thickening with groove sign causing significant dyspnea and hypoxia. **(C)** Fibrosing changes in the deep reticular dermis and full-thickness subcutaneous tissue extending to the deep edge of the biopsy (H&E, x20). **(D)** Perivascular and interstitial inflammatory infiltrates in the background of sclerotic subcutaneous tissue (H&E, x100). **(E)** inflammatory infiltrates, predominantly composed of lymphocytes with occasional eosinophils, (H&E, x200).

He was admitted to a hospital in April 2024 with a 1-week history of worsening confusion and extremity weakness. CT head showed a subdural hematoma causing mass effect and midline shift requiring left-sided mini craniotomy. Mild eosinophilia (0.65 × 10^9^/L) was noted on bloodwork. Avelumab immunotherapy was held and was not restarted. One month later (May 2024), the patient experienced progressive skin thickening involving his bilateral (BL) forearms, thighs, and abdomen while sparing his hands and feet. Physical examination displayed a positive “groove sign” (**Figure 1A**). He was assessed by rheumatology with repeat bloodwork (June 2024) revealing normal erythrocyte sedimentation rate (ESR), C-reactive protein (CRP), hemoglobin, eosinophils, creatinine, liver function test (LFT), and mild thrombocytopenia (116 × 10^9^/L). Anti-nuclear antibody (ANA) and scleroderma antibody panel were not requested. Right forearm skin biopsy (June 2024) was reviewed by dermatopathology showing sclerosis of the deep dermis and full-thickness subcutis, with involvement by lymphocytic infiltrate and occasional eosinophils, suggesting EF (**Figures 1C–E**). MRI of the axial skeleton (July 2024) demonstrated BL forearm and leg edema concerning for myopathy without definitive fascial thickening or peri-fascial edema. From this information, a diagnosis of EF was established.

**Figure 2:**
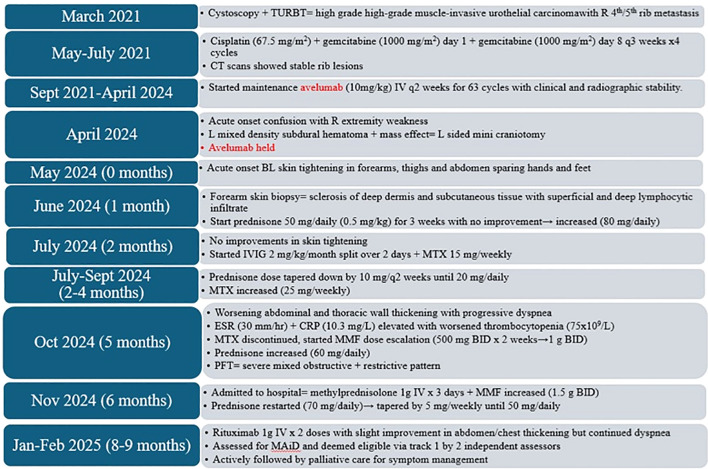
Treatment course for case 1. Timeline in months from onset of eosinophilic fasciitis. TURBT, trans-urethral resection of bladder tumour; R, right; L, left; q, every; CT, computer tomography; BL, bilateral; IVIG, intravenous immunoglobulin; MTX, methotrexate; ESR, erythrocyte sedimentation rate; CRP, C-reactive protein: MMF, mycophenolate; BID, twice a day; PFT, pulmonary function test; MAiD, medical assistance in dying.

Prednisone 50 mg daily (0.5 mg/kg) for 3 weeks was prescribed with no improvement, with an increase to 80 mg daily at the end of June 2024. In July 2024, IVIG 2 mg/kg/month split over 2 days and methotrexate (MTX) 15 mg weekly were initiated. Prednisone dose was tapered by 10 mg every 2 weeks (July–September 2024) until 20 mg daily while MTX was increased to 25 mg weekly (Sept 2024). In October 2024, the patient reported progressive worsening of abdominal and thoracic thickening with progressive dyspnea (**Figure 1B**). Blood work revealed elevated ESR (30 mm/h) and CRP (10.3 mg/L), with worsened thrombocytopenia (75 × 10^9^/L) requiring MTX discontinuation. Mycophenolate mofetil (MMF) was started at 500 mg twice daily (BID) for 2 weeks then continued 1 g BID while prednisone was increased back to 60 mg daily (Oct 2024). Pulmonary function tests (PFTs) demonstrated a severe mixed obstructive and restrictive pattern secondary to progressive abdominal and chest restriction, with decreased FEV1/FVC (58.3) and total lung capacity (4.38 L; 56% predicted). CT chest showed no signs of pulmonary fibrosis. Despite escalation of treatment, the patient continued to experience refractory dyspnea and worsening abdominal wall hardening. He was admitted to a hospital (November 2024) for pulse steroids (methylprednisolone 1 g IV for 3 days) and his MMF was increased to 1.5 g BID. These changes slightly improved his abdomen/chest wall skin thickening on examination, but his dyspnea persisted and ultimately the patient was discharged on home oxygen (2 L nasal prong). Immunosuppressive agents at discharge included MMF 1.5 g BID, IVIG 2 mg/kg split over 2 days monthly and prednisone 70 mg daily for 2 weeks with a taper by 5 mg weekly down to 50 mg daily. Despite his pulse steroid, over the next 2 months (December 2024–January 2025), the patient’s dyspnea continued to worsen. He was given rituximab 1 g IV for two doses (January and February 2025) with his treating rheumatologist noting mild clinical disease improvement. Despite some progress, his dyspnea and hypoxia continued to significantly impact the patient’s quality of life. He was evaluated by two medical assistance in dying (MAiD) assessors (February 2025) and deemed eligible for track 1, with his death being “reasonably foreseeable” given the nature of his incurable disease in the setting of an advanced stage of decline. While no date has been set, the patient expressed a desire to retain the option for MAiD moving forward (**Figure 2**). He continues to actively be followed by palliative care with symptom management at this time although continues to be affected daily by his current symptoms that have significantly impacted his quality of life.

### 2.2 Case 2

A 48-year-old previously healthy male patient with no notable past medical history, family history, or medications presented with stage IIIC (T2aN3c) melanoma of the left preauricular area who had an excisional biopsy in November 2017 with wide-local excision and sentinel lymph node biopsy in January 2018. Two out of three preauricular nodes were positive for metastases. BRAF mutation testing was negative. CT chest/abdomen/pelvis, FDG-positron emission tomography (PET) scan, and MRI brain were all negative for metastatic disease. The patient received nivolumab 480 mg IV every 4 weeks for 1 year (February 2018-February 2019). Imaging during and after adjuvant treatment showed no concerns for recurrence. However, 9 months after completing nivolumab (November 2019), he presented to the hospital with a 2-week history of symmetrical BL lower leg and forearm edema, skin tightening, myalgias, and polyarthralgia involving the ankles, knees, elbows, and shoulders. Initial blood work showed normal CK, thyroid function, glucose, creatinine, and LFT with mildly elevated ESR (7 mm/h), CRP (43 mg/L), and eosinophils (1.1 × 10^9^/L). BL lower leg Doppler and a transthoracic echo were negative for deep vein thrombosis and congestive heart failure. Prednisone 40 mg daily (0.5 mg/kg) was subsequently initiated in November 2019 with some improvement in his skin tightening and edema and tapered to 10 mg weekly until completion in late December 2019. Two weeks following prednisone taper, the patient developed recurrence of his symptoms. Repeat bloodwork in January 2020 showed high ESR (27 mm/h) and CRP (59.5 mg/L) with normal CK.

**Figure 3:**
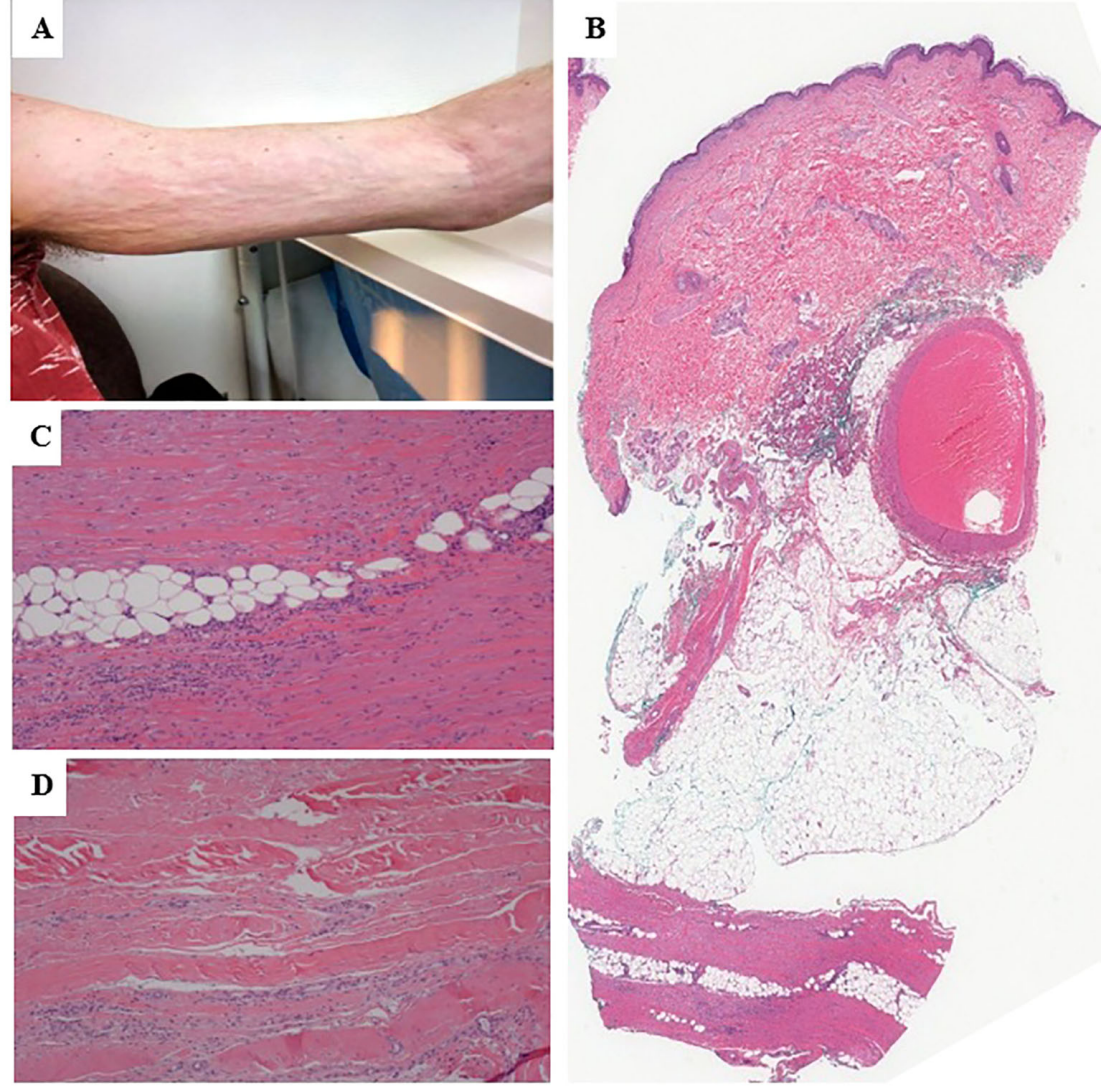
Physical exam findings and H&E staining for case 2- **(A)** Physical exam findings showing groove sign and skin tightening on BL forearms on initial assessment at presentation. **(B)** Full thickness skin biopsy of right forearm showing sclerosis of the deep dermis and subcutaneous tissue (H&E, x0). **(C, D)** Thickening of the fascia with lymphocytic infiltrates consistent with EF (H&E, x20).

Prednisone was restarted at 60 mg daily in February 2020. A rheumatologist noted symmetrical BL skin tightening of forearms and legs sparing hands and feet with a positive groove sign and peau d’orange over BL medial forearms (**Figure 3A**). Serologies showed a positive ANA 1:80 with a speckled pattern. His remaining serologies were otherwise unremarkable including negative antineutrophil cytoplasmic antibodies (ANCA), negative extractable nuclear antigen antibodies (ENA), rheumatoid factor (RF) <10 kIU/L, anti-citrullinated protein antibodies (anti-CCP) <16 units, double-stranded DNA (ds-DNA) <12 IU/mL, normal serum/urine protein electrophoresis (SPEP/UPEP), and normal complement (C3, C4) levels. Right forearm biopsy (February 2020) was reviewed by dermatopathology, which showed fascial fibrosis with lymphocytic infiltrates, occasional plasma cells, and no eosinophils (**Figures 3B–D**). MRI of BL forearms showed skin thickening with deep fascial thickening of both intermuscular/subcutaneous fascia. A diagnosis of EF was considered most likely and high-dose methylprednisolone IV 250 mg for 3 days was given in February 2020 followed by a prednisone taper from 80 mg daily down to 5 mg every 2 weeks. MTX was started at 17.5 mg weekly in March 2020 and increased to 20 mg weekly. In April 2020, he developed progressive BL calf paresthesia and foot drop. He was assessed in a neuromuscular clinic with EMG showing a combination of demyelination and axon loss affecting BL peroneal nerves. With a working diagnosis of immune checkpoint-induced neuropathy and weakness, IVIG (2 mg/kg) was administered over 5 days (June 2020) with no improvement. Repeat EMG (late June 2020) showed BL peroneal neuropathies with conduction blocks at the fibular heads with weakness ultimately felt secondary to significant weight loss rather than immunotherapy.

**Figure 4:**
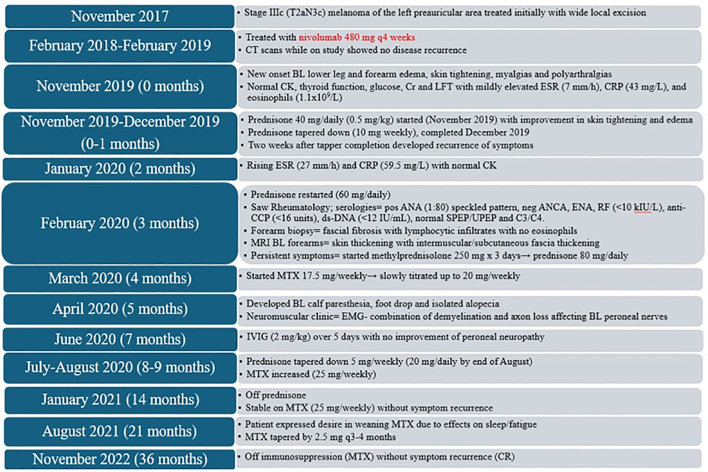
Treatment course for case 2. Timeline in months from onset of eosinophilic fasciitis. q, every; BL, bilateral; CT, computer tomography; CK, creatine kinase; Cr, creatinine; LFT, liver function test; ESR, erythrocyte sedimentation rate; CRP, C-reactive protein; ANA, anti-nuclear antibody; ANCA, antineutrophil cytoplasmic antibody; ENA, extractable nuclear antigen antibodies; RF, rheumatoid factor anti-CCP: anti-citrullinated protein antibodies; ds-DNA, double-stranded DNA, SPEP/UPEP, serum/urine protein electrophoresis; C3/C4, complement 3/4; MRI, magnetic resonance imaging; MTX, methotrexate; EMG, electromyography; IVIG, intravenous immunoglobulin; CR, complete remission.

Between July and August 2020, the patient’s prednisone was slowly tapered down to 20 mg daily and his MTX dose was increased to 25 mg weekly. Despite the tapering dose of prednisone, his symptoms slowly continued to resolve, including his myalgias, arthralgias, lower leg edema, and skin tightening. By October 2020, his prednisone was down to 7.5 mg daily, and by January 2021, he was off steroids and stable on MTX 25 mg weekly. In August 2021, the patient continued weaning MTX, reducing by 2.5 mg every 3–4 months. By November 2022, he was off immunosuppression and had no further concerns for EF flares (**Figure 4**). He had a recurrent left antihelix invasive melanoma resected in November 2022, with pathology consistent with an in-transit recurrence. He received adjuvant radiotherapy (50 Gray in 20 fractions), which was completed in May 2023. PET scan performed in May 2023 showed no evidence of metastatic disease. Subsequent 2 years of follow-up has shown no disease recurrence as of April 2025. Although the patient’s overall recovery did take place over many months, throughout the treatment process, he remained motivated and focused on his recovery. Currently, he is almost back to his pre-EF level of activity and functioning.

## 3 Discussion

Although several case reports have previously documented the occurrence of ICI-induced EF, we report two unique presentations of delayed EF, defined as onset of EF after ICI cessation. Case 1 occurred 1 month following discontinuation of avelumab after 32 months of well-tolerated administration, and Case 2 occurred 9 months after completing 1 year of adjuvant nivolumab. On review, no other inciting events were identified with rheumatology for both patients prior to their initial dermatologic presentation including inciting medications (no PPIs prior to prednisone or statins), environmental exposures (chemical solvents and pesticides), or recent infections (viral, bacterial, or parasitic). Of note, the patient in case 2 did have a paternal uncle with a history of systemic lupus erythematosus, but there was no other personal or family history of rheumatologic disorders or cancers in both patients. These presentations highlight the need to remain vigilant for irAEs even following discontinuation of ICI therapy and are distinct from most ICI-induced EF described during active ICI therapy (7–10). Our two cases met the most widely accepted diagnostic framework for EF, the Pinal-Fernandez criteria, which require either two major criteria or one major plus two minor criteria, while excluding systemic sclerosis (4). Case 1 fulfilled both major and two minor criteria (groove sign and muscle weakness), while Case 2 fulfilled both major and three minor criteria [peripheral eosinophilia (1.1 × 10^9^/L), groove sign/peau d’orange, and MRI evidence of fascial involvement].

Pharmacovigilance data have shown a link between ICIs and EF. One French analysis identified ICIs as the most commonly implicated drug class in EF with an overall reporting odds ratio (ROR) of 75.3 [95% confidence interval (CI): 46.9–120.8], with nivolumab demonstrating the highest association (ROR: 104.3; 95% CI: 61.0–175.0), followed by pembrolizumab and ipilimumab (39.8; 95% CI: 14.6–108.2 and 35.1; 95% CI: 11.1–110.8, respectively) (8). To our knowledge, only one prior case has documented EF post-immunotherapy discontinuation occurring 1 month after pembrolizumab cessation for metastatic melanoma (11). Both of our presented cases of EF had ICI therapy for ≥12 months, and neither experienced irAEs during active treatment. Prior reports show a mean EF onset of 376 days (12.4 months) after ICI initiation (6, 7, 9), although the only case reported with avelumab occurred 1.5 months into treatment (10).

Our EF cases demonstrate an important emerging concept of delayed immune-related events (DIREs), irAEs that occur ≥90 days after ICI cessation. Couey et al. described 23 DIRE cases with a median latency of 6 months post-ICI discontinuation (12). Such events may be underrecognized, as adverse event reporting windows in clinical trials frequently terminate within 90 days of final treatment. A retrospective study found that 15% of patients continuing ICI beyond 12 months developed delayed irAEs, and 54% of these were off therapy at the time of event onset (13). The underlying mechanism of DIREs remains incompletely elucidated. One postulated etiology is loss of immune tolerance. ICIs impair regulatory T-cell function and other immunologic checkpoints, potentially leading to dysregulated immune activation upon a secondary insult such as infection, trauma, or metabolic stress (14). In Case 1, the development of EF shortly after a subdural hematoma suggests that central nervous system trauma may have served as a precipitating event in a patient with a previously ICI-primed immune system. Another proposed mechanism is epitope spreading, a process whereby immune-mediated tissue injury releases novel self-antigens, leading to the activation of autoreactive T or B cells against previously tolerated epitopes (15). This mechanism may help explain the temporal dissociation between ICI exposure and EF onset, as even subclinical inflammation during therapy may subsequently escalate due to persistent autoreactivity, even in the absence of the drug.

In evaluating patients with suspected ICI-induced EF, differential diagnoses include systemic sclerosis, morphea (both linear and generalized), and scleromyxedema. Neither patient exhibited Raynaud’s phenomenon, digital ulcers, or systemic involvement, and serologic markers, including ENA, were negative or not contributory. Overall, these findings reduced the likelihood of systemic sclerosis. EF can also occur as a paraneoplastic syndrome in solid or hematologic malignancy, and a retrospective review conducted at the Mayo Clinic reported a 13.4% risk of associated malignancy among patients with EF, of which a proportion were classified as paraneoplastic EF (16). In both our case reports, there were no clinical or radiographic evidence of cancer recurrence at EF onset, making paraneoplastic EF unlikely.

Management of EF is complex due to its rarity and lack of consensus guidelines. First-line therapy involves glucocorticoids, often requiring high initial doses and a prolonged taper. Second-line agents include MTX, MMF, cyclosporine, IVIG, and biologic agents such as rituximab or infliximab (6, 9, 17, 18). A cohort study of 63 patients with EF reported a 64% complete response rate with MTX and prednisone combination therapy, and a mean steroid duration of 18 months (17). A review of 15 ICI-induced EF cases revealed that 90% were treated with systemic immunosuppression following ICI discontinuation, most commonly prednisone (40–80 mg/day), MMF (3 g/day), methylprednisolone (ranging from 24 mg/day to 1 g/day), MTX (15–20 mg/week), or infliximab (3 mg/kg) (9). A separate study of 32 patients reported a mean prednisone dose of 52 mg/day and better outcomes in those receiving pulse methylprednisolone (500–1,000 mg/day for 3 days) (18).

In our cases, both patients required high-dose prednisone (50–80 mg/day) and prolonged immunosuppression. Both experienced flares following early tapers, requiring escalation. Case 1 was transitioned from MTX to MMF due to thrombocytopenia and required monthly IVIG (2 g/kg over 2 days) and rituximab for progressive disease. Case 2 was treated with MTX 25 mg/week and a short course of IVIG (administered over 5 days), ultimately achieving complete remission over 33 months. Both patients required pulse steroids due to symptom progression. Steroid duration totaled 10 months (Case 1) and 33 months (Case 2) (**Figures 2**, **4**), well above the previously reported mean duration of therapy of 18 months for complete remission in other cases of ICI-induced EF (17). These cases illustrate the importance of slow tapering, close monitoring, early use of steroid-sparing agents, and early subspecialist involvement. Importantly, the initial steroid tapers were likely too rapid in both cases, contributing to symptom recurrence and a more prolonged disease course.

Limitations of these reports include the absence of full serologic evaluation in Case 1, which, while not required for diagnosis, may have further substantiated the autoimmune etiology. Nonetheless, both patients were promptly evaluated, biopsied, and treated, and the diagnoses were confirmed using current criteria. Another limitation was the speed at which the first steroid tappers were conducted, which ultimately led to symptom recurrence, leading to a prolonged treatment course for both patients. This suggests that a slower steroid taper, early subspecialist involvement, and initiation of steroid sparing agents may be beneficial in improving clinical outcomes.

## 4 Conclusion

Recognizing and screening for delayed onset, post-treatment irAEs such as EF is paramount as we continue to expand the scope of ICI treatments for cancer. EF should be considered in patients with current or prior ICI exposure who present with myalgias, arthralgias, extremity edema, and cutaneous thickening, particularly if associated with peau d’orange, groove sign, and sparing of the hands and feet. Diagnostic workup should include eosinophil count, serologies (including ANA and ENA), MRI of involved areas, full-thickness skin biopsy, and consideration of the Pinal-Fernandez criteria. Early recognition of EF, subspecialist involvement, and aggressive treatment with immunosuppression including steroid-sparing agents with slow subsequent taper are important to optimizing outcomes.

## Data Availability

The original contributions presented in the study are included in the article/**Supplementary Material**. Further inquiries can be directed to the corresponding authors.
